# Water-Quality Assessment and Pollution-Risk Early-Warning System Based on Web Crawler Technology and LSTM

**DOI:** 10.3390/ijerph191811818

**Published:** 2022-09-19

**Authors:** Guoliang Guan, Yonggui Wang, Ling Yang, Jinzhao Yue, Qiang Li, Jianyun Lin, Qiang Liu

**Affiliations:** 1 Department of Geography and Information Engineering, China University of Geosciences, Wuhan 430074, China; 2 Ningbo Ligong Environment and Energy Technology Co., Ltd., Ningbo 315800, China; 3 Sichuan Province Environmental Monitoring Station, Chengdu 610091, China

**Keywords:** water quality evaluation, pollution risk, water-quality early-warning system, machine learning, web crawler, LSTM

## Abstract

The openly released and measured data from automatic hydrological and water quality stations in China provide strong data support for water environmental protection management and scientific research. However, current public data on hydrology and water quality only provide real-time data through data tables in a shared page. To excavate the supporting effect of these data on water environmental protection, this paper designs a water-quality-prediction and pollution-risk early-warning system. In this system, crawler technology was used for data collection from public real-time data. Additionally, a modified long short-term memory (LSTM) was adopted to predict the water quality and provide an early warning for pollution risks. According to geographic information technology, this system can show the process of spatial and temporal variations of hydrology and water quality in China. At the same time, the current and future water quality of important monitoring sites can be quickly evaluated and predicted, together with the pollution-risk early warning. The data collected and the water-quality-prediction technique in the system can be shared and used for supporting hydrology and in water quality research and management.

## 1. Introduction

Environmental monitoring is a fundamental component of environmental protection and a key tool for advancing ecological development. China has had an established national surface-water monitoring network since 1991. Since 1999, it has begun to conduct pilot automatic monitoring of the surface-water quality in some river basins [1]. Currently, it has thousands of national surface-water environmental-quality-monitoring sections, forming a national surface-water environmental-quality-monitoring network, which established a monitoring mode. This combines the manual monitoring of collection and separation with the automatic monitoring of water quality, and discloses these real-time monitoring data [2]. It is of great significance to fully dig out the value of real-time monitoring data of national surface-water quality, which provides a better comprehension of the changing trends in China’s water environment [3]. 

With the rapid development of the global network, a large amount of information and data has flooded into the Internet [4]. Understanding how to effectively acquire useful information from network public data has emerged as a main issue. Web crawler technology was created in order to extract the relevant data in batches from the vast amount of data on the Internet automatically, and it is currently widely used in search engines, large-scale data mining and analysis, artificial intelligence and machine learning dataset production, among other fields [5]. A lot of methods have been created for data crawling and gathering from the web, such as the autonomous crawling and analysis tool from the dark web [6], the query-based crawler [7] and the web-crawled literature [8]. It is feasible to readily and swiftly obtain real-time monitoring data from the opened national surface-water quality data through the robust data-crawling, processing and analysis capabilities of a web crawler.

In reality, several researchers are now using the national surface-water-quality-monitoring dataset as a data source and a support for their work. For example, the evolution of water quality in Ma’an City [9], Zhujiang River [10] and Taizi River [11] have been analyzed by crawled data from the open dataset. In recent years, the spatial distribution of river-water quality and its affecting factors at a national level has been conducted based on these data [12]. The aforementioned research demonstrates that, although the national surface-water quality-monitoring dataset has been fully utilized in the assessment of surface-water environmental quality and the analysis of historical water-quality changes, they are rarely used in the field of surface-water quality prediction [13]. In reality, employing historical data to predict and analyze water quality has substantial practical implications for successfully reducing the likelihood of water contamination incidents, assisting with environmental management and protection, etc. Massive, high-frequency and real-time monitoring data should not only participate in the evaluation, assessment and ranking of surface-water environmental quality, but it should also be applied to the early warning and prediction of water environmental quality [14,15]. Water-quality assessment and prediction are the basis of water-pollution prevention, as well as control and environmental management. Meanwhile, the statistics on water quality in the entire nation and in various regions are currently missing from China’s public datasets on water quality, and the visualization quality is subpar. On the other hand, forecasting capabilities are lacking in the current national data sharing system for water quality monitoring.

Numerical models are used extensively in water quality prediction and early warning [16,17]. However, numerical models need a lot of condition data, which adds a lot of labor. Artificial neural networks (ANNs) have the following characteristics: strong nonlinear mapping ability, high learning accuracy and strong robustness. They represent a better modeling method for water quality prediction nationwide. Among these, long short-term memory (LSTM) networks have a good performance in processing data with time series characteristics [18]. They addresses the issues of gradient explosions and the gradient disappearance of conventional recurrent networks; LSTM networks are presently used in many fields, such as trend predictions of COVID-19 [19], sudden change simulations in financial markets [20] and water quality prediction [21,22]. This research has verified that the LSTM model has better prediction accuracy than the traditional numerical models. The LSTM neural network has been utilized extensively in the field of water environment, although the majority of these networks are only employed in theoretical research projects and cannot be integrated with monitoring data for real-time prediction, which has no practical application. In fact, pollution control, environmental governance and environmental protection all benefit significantly from real-time prediction using monitoring data. As a result, it is necessary to build a real-time water-quality-prediction system with the help of the LSTM neural network.

Artificial neural network technology has been widely used in the field of environmental management and prevention. Developing a real-time monitoring and early-warning system by fusing it with water quality monitoring with the aid of the neural network’s potent learning capabilities and the help of the powerful learning ability of the neural network is a popular research issue. In fact, a number of established water-quality-prediction and early-warning systems have surfaced both domestically and internationally [23]. However, the majority of these systems concentrate on water quality monitoring and early warnings in local water and economic breeding fields, rather than establishing a national water-quality-prediction and early-warning system. Therefore, this paper constructs a water-quality-prediction and early-warning system, using web crawler technology to obtain real-time monitoring data from national surface-water monitoring stations and combining the LSTM neural network model to provide water-quality-prediction and early-warning services. Comparatively speaking, this system makes full and effective use of the national water-quality-monitoring data and establishes a relatively complete national water-quality-prediction and early-warning system, as well as a data sharing system, which provides help for the prevention and control of national water environmental pollution and environmental management.

## 2. Data and Methods

### 2.1. Data Sources

The hydrological and water-quality data mentioned in this article were collected from the national water and rain information network of the Ministry of Water Resources of the People’s Republic of China (http://xxfb.mwr.cn/sq_djdh.html accessed on 16 September 2022) and the national surface-water quality monitoring and publishing system of the Ministry of Ecology and Environment of the People’s Republic of China (https://szzdjc.cnemc.cn:8070/GJZ/Business/Publish/Main.html accessed on 16 September 2022).

### 2.2. The Philosophy of This System

**Figure 1:**
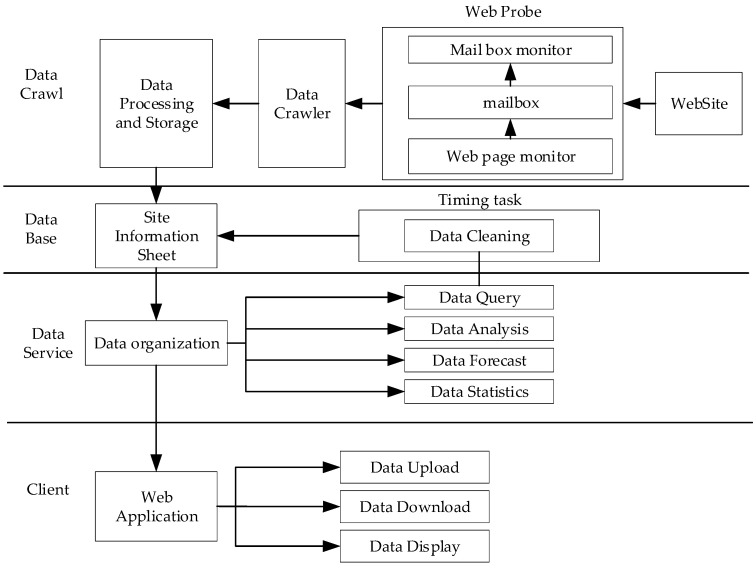
The philosophy of water-quality-prediction and early-warning system.

The purpose of this system was to develop functions for data crawling, storage, water quality prediction and early warnings based on the public hydrology and water-quality dataset. One of the most significant features in this process was the ability to automatically obtain public hydrology and water-quality data through data collecting, cleaning and storing in real time. On this basis, the LSTM model could be constructed and trained to accomplish the function of water quality prediction and early warnings. The philosophy of this system is shown in Figure 1.

#### 2.2.1. Data Crawling

A monitoring script was set up in the target website through the distill web monitor plug-in provided by the browser. Crawlers will be launched to acquire and store data automatically once the update of the target website is detected.

#### 2.2.2. Database

In this system, we chose MongoDB as the storage database for data storage and maintenance functions, and to establish the data table shown in Table 1.

Additionally, it also provided a data-cleaning service to remove redundant data and store new data.

#### 2.2.3. Model Service

The data services section provided data manipulation functions. It was used to establish the connection session between the server and client, retrieve and analyze data from the database according to certain conditions, and return prediction results of water quality by using the LSTM neural network model.

#### 2.2.4. Client

This module provided the functions of data interaction, including data upload, data download, rendering and producing displays through words, tables or charts. Users could log in to the system through a browser and make the related operations.

### 2.3. Process of Data Collecting by the Web Crawler

**Figure 2:**
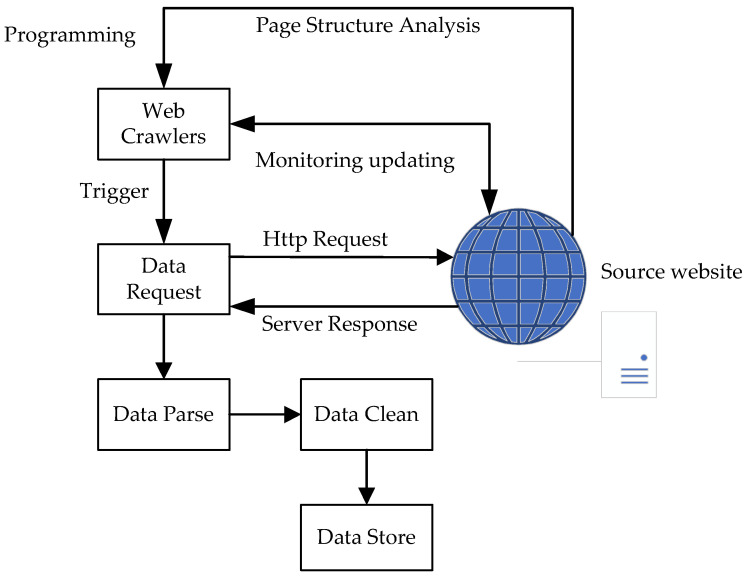
Process of data collecting by web crawler used in the system.

Web crawlers were used to periodically collect real-time monitoring data of water-quantity- and water-quality-monitoring stations. The process of the crawler is shown in Figure 2. 

#### 2.3.1. Website Monitoring and Crawler Starting

A monitor mechanism was created to monitor the content change of the national water and rain information network, and the national surface-water-quality-monitoring and publishing system through the Distill Web Monitor plug-in provided by the browser. An e-mail is sent to a specified mailbox if there are some data updates. The measurement time, used for data update judgment, of water quality data is monitored by Xpath syntax. The crawler starts once an update e-mail is captured and parsed by a mailbox-monitoring script.

#### 2.3.2. Data Crawling

The URL of the target website is first parsed through the network data-catching tool after launching the crawler. Then, a POST request is sent to the object URL through the request library in Python. After that, the browser receives a response in JSON data from the object URL. Water quality data is obtained from the JSON data.

#### 2.3.3. Data Cleaning and Storing

Data cleaning is essential before data is stored in a database. After obtaining the water quality data from the crawler module, the next step is to optimize them. The repeating data are deleted, as well as the null data. After that, the data are stored in MongoDB, which is accomplished by calling PyMongo through Python. Firstly, query the monitoring time of the site and estimate whether the current monitoring time is the same as that stored in the database table. If so, it can be considered that the data under the corresponding site was crawled and updated. The storing step will not be started. Otherwise, update the corresponding site data with the new crawled and cleaned data. Secondly, if the current traversal site is not recorded in the site data table, create corresponding site records in the site data table and store the data.

### 2.4. Model Services Development for Water Quality Assessment and Early Warnings Based on the LSTM Neural Network

**Figure 3:**
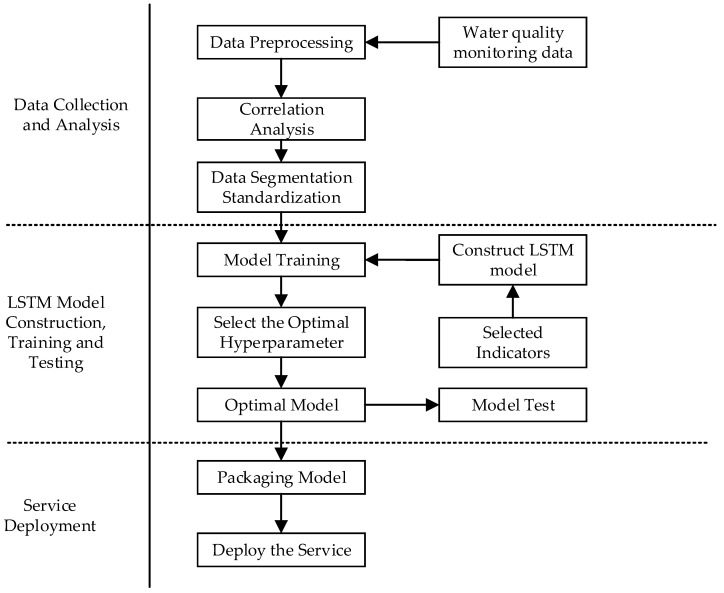
Model construction process of water quality prediction based on LSTM.

The long short-term memory (LSTM) network is an improved and solvable special RNN model [24]. By introducing a new internal state and gate mechanism, LSTM networks have a good performance in solving the problems of gradient disappearance and long-term dependence, which makes LSTM have selective memory functions [25]. The construction process of the water-quality-prediction model for river pollution accidents based on LSTM is shown in Figure 3. 

#### 2.4.1. Dataset Organization and Analysis

The dataset contains water quality data from 1863 key monitored sections in China. The first step is to pretreat the obtained data. The input variables corresponding to the model output variables are determined through correlation analysis. After that, the dataset is divided into the training set, test set and validation set. Then, all of these data are standardized.

#### 2.4.2. Model Developed by LSTM

**Figure 4:**
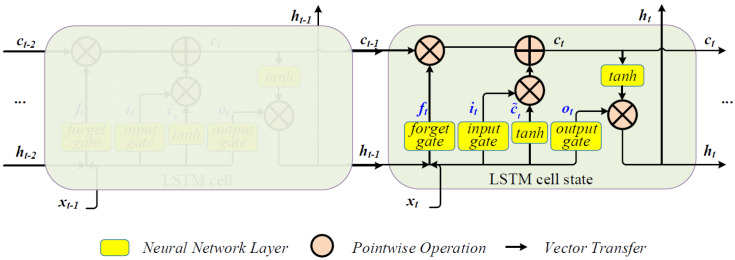
Structure of the LSTM model.

The LSTM model is an advanced recurrent neural network (RNN) that includes specialized memory blocks for capturing multi-timestep relationships [26]. The model structure used in this system is shown in Figure 4.

Where $c_{t-1}$ is the cell state at time *t*−1, $h_{t-1}$ is the output at time *t*−1, $x_{t}$ are new inputs at time t, $o_{t}$ is the value that the output gate produces to determine which parts of the cell state to output, tanh denotes the *tanh* function, $\tilde{c_{t}}$ is the candidate cell state and *i_t_* is the coefficient of $\tilde{c_{t}}.$

The LSTM cell state is the memory space of the whole model, which changes over time. Three control gates, namely the neural network layer, are the core modules controlling what information is transferred. In the system, the LSTM model was built based on the TensorFlow framework. Water quality parameters, such as pH, ammonia nitrogen ($NH_{4}^{+}$), dissolved oxygen (DO), water temperature (WT), potassium permanganate index (COD_Mn_) and turbidity (TU), are set for water environment evaluation. Future water quality parameters at time *t*+1 are simulated by LSTM using the historical value of water quality parameters. The whole dataset in China is divided to 10 watersheds. Every watershed is divided into three parts: upstream, middle, and downstream sub-watershed. Every sub-watershed is modeled by a single LSTM model. Every water quality index has its own LSTM model. There are 210 developed LSTM models in the whole of China. 

#### 2.4.3. Model Training and Testing

After the model is built, the training set of these data is put into the LSTM model for model training. Optimal hyperparameters parameters are then determined according to the verification based on the model validation dataset. Finally, the test model accuracy performance of water quality prediction is made on the test set. 

#### 2.4.4. Model Services Deployment

After model testing, the adequate model is picked as a TensorFlow Server to the system as a model service.

### 2.5. Client Structures

**Figure 5:**
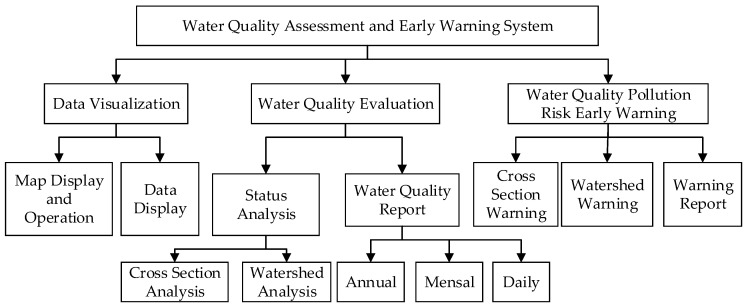
The submodules and functions designed in the client.

In order to fulfill the need for a water-quality-assessment and pollution-risk early-warning system, the client module was designed to include a data visualization module, a water quality evaluation module and a water-quality pollution-risk early-warning module, as shown in Figure 5.

#### 2.5.1. Data Visualization Module

This module is mainly used to visualize the spatial location information of water-quality-monitoring stations, data analysis and prediction results. Two important functions are designed in the data visualization module. For map display and operation, the GeoServer and webGIS are used to build a GIS map, which provides the function for map display, query and spatial data rendering. For data visualization, the time series data is visualized in the form of a table, chart and animation.

#### 2.5.2. Water-Quality-Assessment Module

This module is mainly used to integrate and analyze the current water-quality-monitoring data and generate water quality reports within a certain time range, according to the historical monitoring data. The function of the current water quality analysis is developed. The water-quality-attainment-standard analysis, proportion of water quality categories analysis and the link relative ratio analysis are provided, which allows users to retrieve any site by watershed name, province name or cross-section name. Meanwhile, water quality reports can be made automatically according to the user’s demands. The annual water quality report, monthly water quality report and customized definition report can be made after determining the parameters, time range and evaluation methods selected by users.

#### 2.5.3. Water-Quality-Prediction and Early-Warning Module

This module is mainly used to forecast the tendency of water quality in a specified cross-section or watershed through LSTM. An early warning is made based on the prediction result. The indicators of prediction can be selected from water quality indexes. The prediction module has two kinds of water-quality-prediction and early-warning modes. 

Firstly, there is the automatic prediction and early warning: The water quality concentration and water quality grade for every water quality station, sub-watershed and watershed are made automatically for 24 h at intervals of one hour. The function is automatically started at 0:00 every day. The detail information of water quality trends can be obtained via the form of a table, chart, etc. The system raises a warning in time once the predicted indicator is over the standard. At the same time, a water quality report containing water quality station and watershed information whose water quality is unqualified is made and sent to the managers.

Secondly, there is the manual prediction and early warning: The system provides a user-defined prediction function. Based on user-specified water-quality parameters, stations and time periods, the LSTM model is launched. The prediction results, as well as early warning reports, are made.

## 3. Results and Discussion

### 3.1. Datasets That Were Crawled

#### 3.1.1. The Spatial Distribution of Monitoring Stations

**Figure 6:**
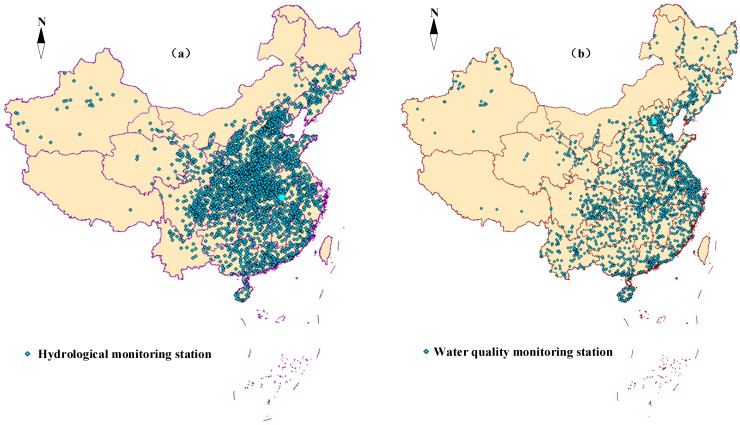
Distribution of hydrological monitoring stations (**a**) and water-quality-monitoring stations (**b**).

There are a total of 2591 hydrological monitoring stations and 3785 national surface-water quality stations obtained in the crawler. The distribution of the data sites crawled is shown in Figure 6.

As can be seen in Figure 6, hydrological stations are mainly distributed in the eastern basin, and hydrological stations in the Yangtze River basin and the Yellow River basin are densely distributed. Hydrological stations are less distributed in the west than the east. Similar to hydrology stations, water quality stations are mainly distributed in the east of China, especially in the Yangtze River basin. On the whole, compared to most research, the location density of sites used for water quality prediction is better [12,27]. This means that the hydrological stations and water quality stations cover most of the water systems in China, and this reflects the characteristics of hydrology and water quality in China well.

Obviously, this dataset contains most of China’s central, southern and eastern regions, and basically covers the main river basins in China. Features of hydrology data indicators and water quality data indicators obtained in the system are shown in Table 2.

#### 3.1.2. The Temporal Distribution of Monitoring Stations

**Figure 7:**
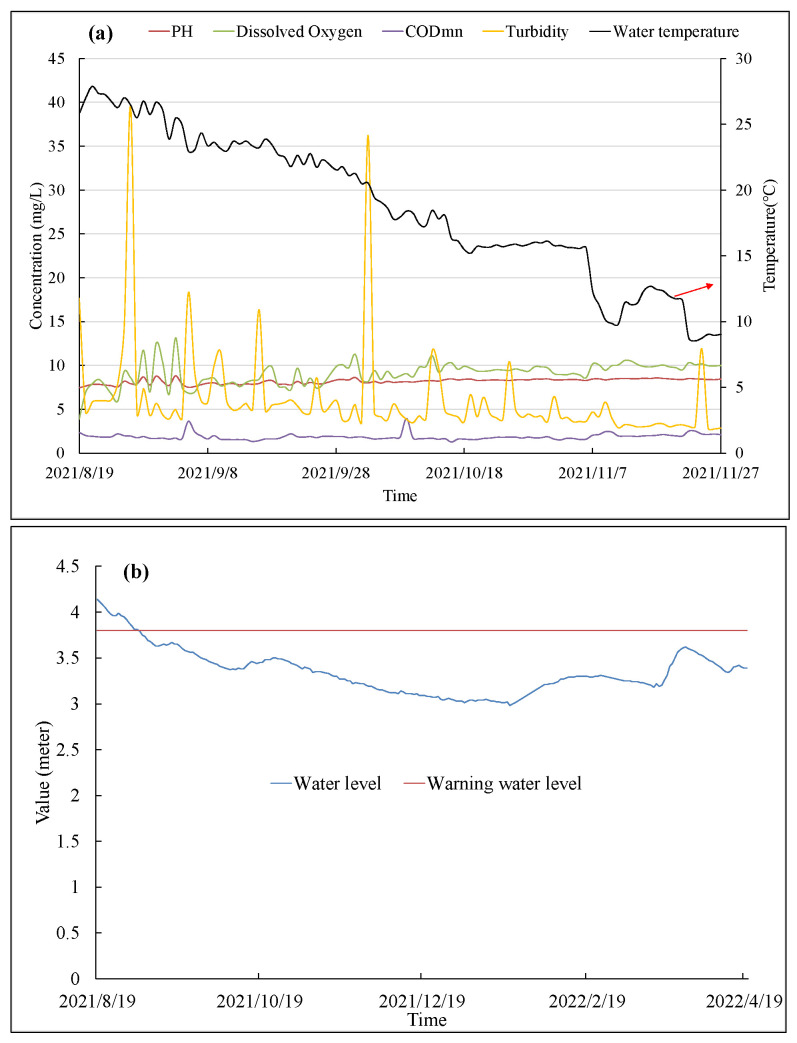
Part of the hydrological monitoring data at Taihu station (**a**) and water quality of North Moat station (**b**).

Hydrological monitoring data from 2005 to today were obtained. These data were published at 8:00 and 14:00. Additionally, water quality created every four hours since 2000 was gained. Monitoring data from two stations was displayed as an example. A part of the hydrological monitoring data from the Taihu station and the water quality of Beijing’s North Moat station are shown in Figure 7.

As shown in Figure 7a, the water stage, warning water state and quantification of flow will be released. It is shown that the water stage in the Taihu station was stable at around 3 to 4 m. For water quality, as is shown in Figure 7b, the concentration of PH, COD_Mn_ and dissolved oxygen in the North Moat station was stable. Meanwhile, the turbidity had relatively large fluctuations from 17 September 2021 to 21 September 2021. These characteristics are similar to other published research [28,29]. Overall, the quality of these datasets is good enough to support water quality prediction.

### 3.2. LSTM Model Training and Validation

**Figure 8:**
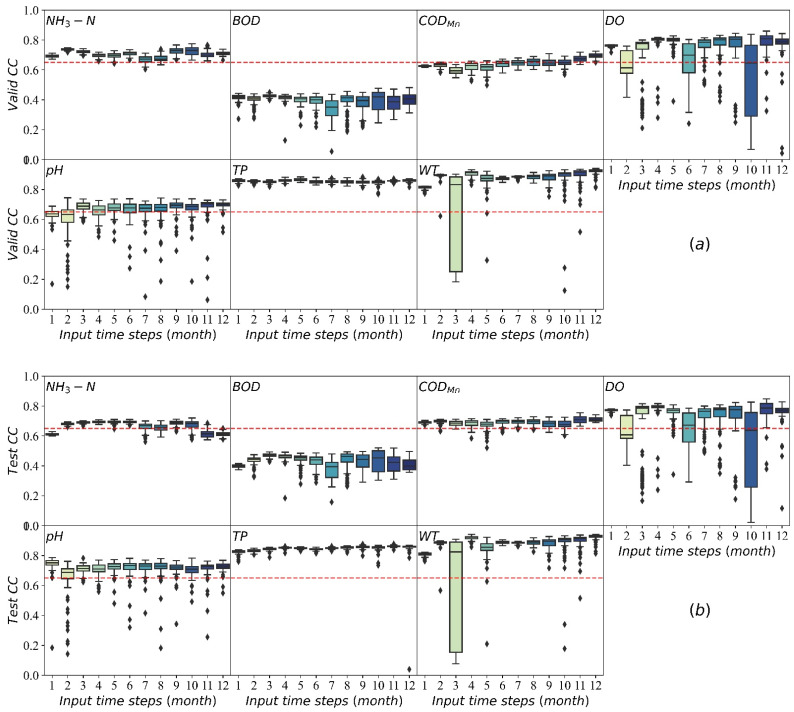
Distribution of cross-correlation (*CC*) values between predicted results and measured values for each water quality indicator predicted results in the the validation set (**a**) and test set (**b**). The lower boundary of the box represents the minimum value. The upper boundary of the box represents the maximum value. The red dotted lines represent the threshold of acceptable model performance (*CC* = 0.65).

LSTM models were trained and validated based on the water-quality-monitoring dataset of every sub-watershed. The Nash efficiency coefficient (NSE) of LSTM models used in this system for water quality prediction in the Haihe River basin can be seen in our published paper [26]. It verified that the LSTMs present are better predictors of BOD, COD_Mn_, CODCr and TP (median Nash–Sutcliffe efficiency reaching 0.766, 0.835, 0.837, and 0.711, respectively) than of ${\mathrm{NH}}_{4}^{+}$, DO, and pH (median Nash–Sutcliffe efficiency of 0.638, 0.625, and 0.229. As an expert for PH (for the cause of large missing PH monitoring data), these models perform very well. Additionally, the LSTM model in the Yangtze River basin can be used as an example. In this dataset, we collected the water-quality-monitoring data of seven indicators in 22 sites from January 2003 to December 2018. The dataset was divided into the training set, test set and validation set. Part of the results of LSTM developed for the Yangtze River basin are shown in Figure 8. *CC* stands for cross-correlation that indicates the effect of simulation, which can be calculated as follows:

(1) $$\begin{array}{c} CC\left(x,y,k\right)=CCF\left(x_{t},y_{t-k}\right) \end{array}$$

(2) $$r\left(x,\;y\right)=\frac{\sum_{i=1}^{n}\left(x_{i}-\bar{x}\right)\left(y_{i}-\bar{y}\right)}{\sqrt{\sum_{i=1}^{n}{\left(x_{i}-\bar{x}\right)}^{2}\sum_{i=1}^{n}{\left(y_{i}-\bar{y}\right)}^{2}}}$$

(3) $$CS\left(x,\;y\right)=\frac{x^{T}y}{\left\|x\|\|y\right\|}$$

(4) $$dCor\left(x,\;y\right)=\left\{\begin{array}{c} \;\sqrt{\frac{dCov{\left(x,\;y\right)}^{2}}{\sqrt{dCov{\left(x\right)}^{2}dCov{\left(y\right)}^{2}}}}\;dCov{\left(x\right)}^{2}dCov{\left(y\right)}^{2}>0\\ \;0\;dCov{\left(x\right)}^{2}dCov{\left(y\right)}^{2}=0 \end{array}\right.$$

(5) $$MIC=\max\left\{\frac{I\left(x,y\right)}{\log_{2}\min\left(n_{x},\;n_{y}\right)}\right\}$$

(6) $$I\left(x,y\right)=H\left(x\right)+H\left(y\right)-H\left(x,y\right)$$

where *CCF* denotes the correlation metric formula (Equations (1)–(5)); *k* is the lag time ranging from 1 to 12; *n* is the number of variables for the simulation or measured data; *x*, *y* are two different water quality variables; $x_{t}=\left\{x_{k},x_{k+1},\;x_{k+2},\;\ldots ,x_{t}\right\}$, $x_{t-k}=\left\{x_{0},x_{1},\;x_{2},\;\ldots ,x_{t-k}\right\}$ and $y_{t-k}=\left\{y_{0},y_{1},\;y_{2},\;\ldots ,y_{t-k}\right\}$;$x=\left\{x_{1},\;x_{2},\;\ldots ,x_{n}\right\}$ and $y=\left\{y_{1},\;y_{2},\;\ldots ,y_{n}\right\}$; $dCov\left(x\right)$ represents the Distance variance of x; $dCov\left(x,\;y\right)$ denotes the Distance covariance between x and y; $H\left(x\right)$ and $H\left(y\right)$ are the entropy of x and y, respectively, and $H\left(x,y\right)$ is their joint entropy; *B(n)* is a coefficient of *n*; $n_{x}n_{y}<B\left(n\right)$ and $B\left(n\right)=n^{0.6}$. 

It is shown in Figure 8 that the *CC* value of the models of the validation set (part a)and test set (part b) is used for predicting ammonia nitrogen ($NH_{4}^{+}$), biochemical oxygen demand (BOD), permanganate index (COD_Mn_), dissolved oxygen (DO), hydrogen ion concentration (PH), total phosphorus (TP) and water temperature (WT). In the test set, models used to predict PH, WT and TP are acceptable since most of their *CC* values are bigger than 0.65, and some models performed well (*CC* > 0.75). Additionally, the *CC*s for $NH_{4}^{+}$ and COD_Mn_ are close to 0.65. In the validation set, the *CC* of six indexes, COD_Mn_, TP, $NH_{4}^{+}$, WT, PH and DO, reach 0.65. Only the *CC*s for BOD are less than 0.65, which is around 0.4. The BOD is measured by the microbial electrode method and is only suitable for low concentrations of water. Meanwhile, the BOD in most of the test sections is relatively large, which makes the measured data fluctuate greatly and without obvious regularity. As a result, it is difficult to predict accurately.

Overall, these results are similar to or even better than the results of published research for monthly water quality predictions [30,31]. According to the previous research, the model is acceptable when the *CC* ≥ 0.65 [32] and very good when the *CC* ≥ 0.75 [26]. Accordingly, the model has a good performance in predicting water quality.

### 3.3. System Developments and Implementation

#### 3.3.1. System Development Environment

Based on the B/S architecture and the model-template-view (MTV) framework for Django 1.8 and Pycharm 2021.3 professional software, the integrated development environment was built. MongoBD was set as the primary database to provide a data storage service. The TensorFlow framework was used to train, build and package the LSTM neural network model to be a TensorFlow Server. 

#### 3.3.2. Functions of the System

**Figure 9:**
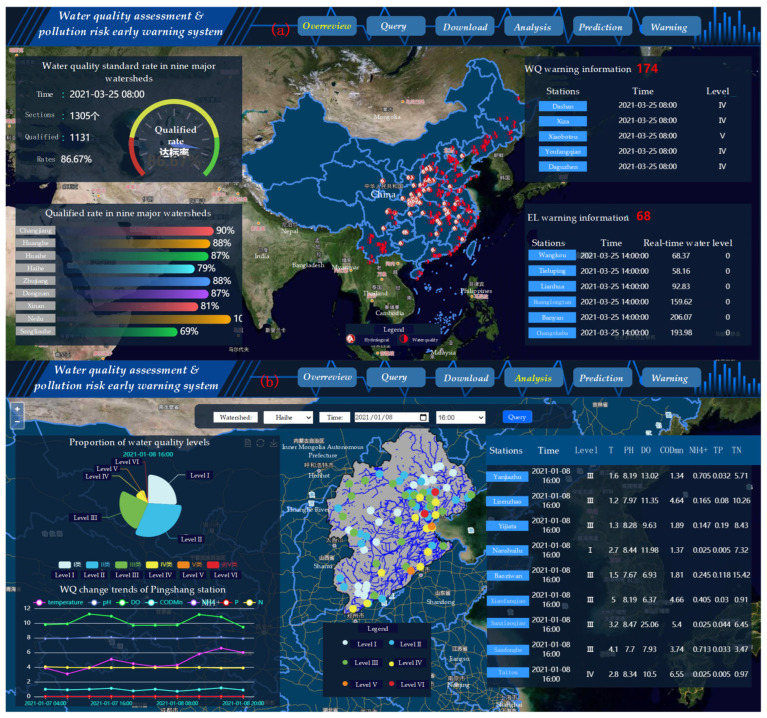
Functions of the water environment data sharing and analysis system ((**a**): overview of water-environment-monitoring data, (**b**): water quality analysis). Level VI of water quality means the water quality is worse than level V.

These functions, such as water-environment-monitoring data visualization, water quality evaluation, water quality prediction and water-pollution-risk early warning were developed in the system (as shown in Figure 9a,b).

(1) Overview of water environment monitoring data

Figure 9a shows the visualization function of water-quality-monitoring data. The upper part of the interface integrates the system function area. In the GIS map, we chose the image map of the Tiandi map service to implement the function of basic map display (https://www.tianditu.gov.cn/ accessed on 16 September 2020). In addition, the translucent and suspended window was used to display the hydrology water-quality-section alarm information. The water quality statistics were set to enhance interactivity with a GIS map.

(2) Water analysis with with water quality evaluation and prediction

Figure 9b shows the general situation of all water quality sections in the Haihe River basin and the chart of trends in water quality indicators of specific sections that are predicted the next day. The suspension toolbar integrates all options for the prediction of water quality of the watershed. It describes the summary of each cross-section and water quality trend, specifying the cross-section in the form of a table, chart, etc., which better reflects the hierarchical relationship between the watershed and section data.

### 3.4. Effect of a Case of Pollution Warning

**Figure 10:**
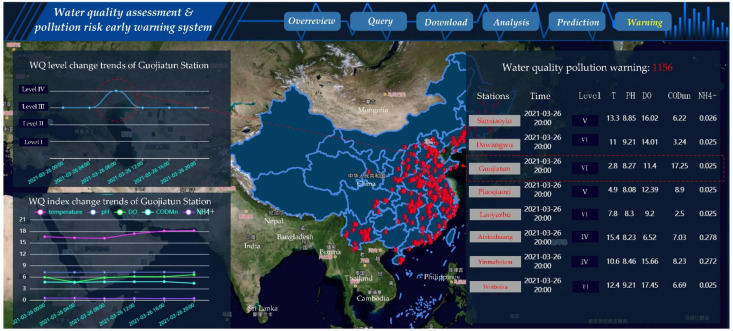
The water quality of pollution waring of prediction results.

The system automatically evaluates the water quality of all sites after prediction by the LSTM neural network model and renders a red point on the GIS map if the water quality is over the standard. As shown in Figure 10, the water quality (WQ) level of the Guojiatun station reached level IV on 26 March 2021 at 4:00, and then reached an inferior level V at 20:00. This means that there may be sudden pollution spills around this station. After the system detected the pollution, the alarm information of the WQ was displayed in the right column of the system. Meanwhile, a warning report of water pollution was generated and sent to the administrator’s mailbox.

In the early 1990s, the concept of an integrated energy and water quality management system (EWQMS) was developed to solve water quality, water supply and energy management problems, simultaneously. Since then, a lot of water quality management systems have been developed to display, monitor and assess water quality. From early displays of water-quality-monitoring data to water quality simulation and prediction with numerical models, water quality management systems have become more and more powerful [33,34]. Previously, we designed a water environment management system with flexible and extensible service-oriented architecture with the data center, system control center, model center and client center based on numerical models. This makes it possible for water quality forecasting in an automatic mode and a user-defined mode [35]. Meanwhile, as well as other water management systems, the previous systems make water quality simulations based on mechanism models. It needs preconcert development and a lot of basic condition data, such as topography data, geomorphology data, hydrology and water pollution source data. The system built in this paper can make water quality predictions rapidly, based on a small number of water-quality-concentration data.

## 4. Conclusions

This paper designed and developed a kind of water-quality-forecasting and warning system. The system can provide functions for data querying, presenting and analyzing, as well as the retrieval and data sharing of historical data. The system can especially be used for water quality assessment and prediction based on LSTM models. It shows great significance for the water and environmental management department in obtaining water quality data and guarding water pollution. The water-quality-prediction and early-warning system constructed in this paper plays an important role in the promotion of environmental monitoring and monitoring work, the building of environmental-monitoring big data systems, and the promotion of ecological civilization construction and ecological environment-monitoring reform.

However, there are still some weaknesses in this system, such as imperfect data sharing, and a lack of verification of the accurate prediction and evaluation results. With the increase in monitoring data and the development of artificial neural networks, this system develops towards a faster, more professional, more reliable and lower-cost direction in the field of water quality monitoring and predicting.

## Figures and Tables

**Table 1:** The functions of tables used in the database.

ID	Tables in the Database	Functions
1	Hydrology and water quality station information table	Store the names and geographical coordinates of hydrology and water quality stations in China
2	Hydrology station table	Store the names of the hydrology sites crawled
3	Water quality site table	Store the names of the water quality sites crawled
4	Hydrological data table	Store the hydrological data crawled
5	Water quality data table	Store the water quality data crawled

**Table 2:** Features of the obtained data in the system.

Category	Variable	Indexes Name	Time Range	Units
Hydrological monitoring data	WL	Water level	Since 2005	m
	WWL	Warning water level		m
	Q	Flow		m³/s
Water-quality-monitoring data	Tub	Turbidity of water	Since 2000	NTU
	COD_MN_	Permanganate index		mg/L
	$NH_{4}^{+}$	Ammonia nitrogen index		mg/L
	TP	Total phosphorus index		mg/L
	TN	Total nitrogen index		mg/L
	PH	Pondus Hydrogenii		-
	CHL	Chlorophyll α content		mg/L
	CA	Algal density of water		cells/L
	BOD5	Biochemical oxygen demand		mg/L
	DO	Dissolved oxygen		mg/L
	WT	Water temperature		°C
	WQC	Water quality classification		-

## Data Availability

The data presented in this study are available upon request from the corresponding author. The data are not publicly available because of privacy concerns.
